# Preventive Effect of a Postbiotic and Prebiotic Mixture in a Rat Model of Early Life Rotavirus Induced-Diarrhea

**DOI:** 10.3390/nu14061163

**Published:** 2022-03-10

**Authors:** Carla Morales-Ferré, Ignasi Azagra-Boronat, Malén Massot-Cladera, Sebastian Tims, Karen Knipping, Johan Garssen, Jan Knol, Àngels Franch, Margarida Castell, Francisco J. Pérez-Cano, María J. Rodríguez-Lagunas

**Affiliations:** 1 Physiology Section, Department of Biochemistry and Physiology, Faculty of Pharmacy and Food Science, University of Barcelona (UB), 08028 Barcelona, Spain; carla.moralesferre@ub.edu (C.M.-F.); ignasiazagra@ub.edu (I.A.-B.); malen.massot@ub.edu (M.M.-C.); angelsfranch@ub.edu (À.F.); margaridacastell@ub.edu (M.C.); mjrodriguez@ub.edu (M.J.R.-L.); 2 Nutrition and Food Safety Research Institute (INSA-UB), 08921 Santa Coloma de Gramenet, Spain; 3 Danone Nutricia Research, 3584 CT Utrecht, The Netherlands; sebastian.tims@danone.com (S.T.); karen.knipping@danone.com (K.K.); johan.garssen@danone.com (J.G.); jan.knol@danone.com (J.K.); 4 Division of Pharmacology, Faculty of Science, Utrecht Institute for Pharmaceutical Sciences, Utrecht University, 3584 CA Utrecht, The Netherlands; 5 Laboratory of Microbiology, Wageningen University, 6708 PB Wageningen, The Netherlands

**Keywords:** Lactofidus, scGOS/lcFOS, rotavirus, suckling rats, microbiota

## Abstract

Rotavirus (RV) is the main cause of gastroenteritis in children. Prebiotics and, more recently, postbiotics are used for preventing and treating gastrointestinal infections. The aim of this study was to analyze the effects of a Lactofidus^TM^, short-chain galacto-oligosaccharides (scGOS) and long-chain fructo-oligosaccharides (lcFOS) mixture, and their combination on RV infection, in a rat model, for early life diarrhea. Fifteen litters of suckling rats were intragastrically administered daily with the vehicle, the prebiotic mixture, the postbiotic or the combination. The RV was inoculated on day 5 and then fecal samples were clinically evaluated daily. Viral shedding, intestinal permeability assay, in vitro blocking assay, immunoglobulin profiles, and anti-RV response were assessed at day 8 and 16 of life. Cecal microbiota composition, intestinal gene expression, and short chain fatty acids (SCFAs) were analyzed at day 16. The incidence and severity of diarrhea were significantly reduced by all the supplementations. Moreover, they showed blocking activity, changes in the immunoglobulin profiles, in gut microbiota, and in the intestinal gene expression. The prebiotic mixture reduced gut permeability and changed the SCFA profile, whereas the postbiotic enhanced the expression of Toll-like receptors (TLRs). The combination preserved most of the individual observed effects, and furthermore, complementary effects, such as an increase in white blood cells and lymphocytes recruitment, as well as upregulation of TLR7 and TLR9 gene expression.

## 1. Introduction

Rotavirus (RV) is a nonenveloped virus of the family *Reoviridae* that infects the enterocytes of the small intestine, causing diarrhea, vomiting, and fever in children and in young animals, including calves and piglets [1,2,3]. In humans, the RV is the leading worldwide etiological agent of gastroenteritis and is responsible for approximately 20–30% of all cases that require treatment in hospitals [4]. No specific curative treatment exists for RV gastroenteritis, and the most common treatment is oral rehydration solutions [1,5]. RV infection can be prevented by vaccination; however, its efficacy is lower in some African and Asian countries [4]. Dietary management is important in the care of infants with acute diarrhea. In countries where malnutrition is common, zinc supplementation can improve the outcome of acute diarrhea [1]. The improvement of hygiene and sanitation conditions reduced the morbidity and mortality due to RV infection [4].

Human milk contains oligosaccharides and glycoproteins that provide protection against microorganisms’ invasion [6]. Moreover, human milk is a source of commensal bacteria to the infant gut that could have immunomodulatory, anti-infectious, and metabolic roles [7]. However, in some cases, breastfeeding is not possible, and infant formula is the substitute for the baby’s nutrition. Therefore, infant formulas try to mimic the composition of breast milk by adding prebiotics, probiotics, and recently, by paying attention to postbiotics [6,8,9].

Prebiotics are defined as a substrate that is selectively utilized by host microorganisms conferring a health benefit [10]. In this sense, human milk oligosaccharides (HMOs) are the main dietary oligosaccharide structures with prebiotic effects in human milk. In infant formula, short-chain galacto-oligosaccharides (scGOS) and long-chain fructo-oligosaccharides (lcFOS) are usually added [9]. Preclinical and clinical studies show that scGOS/lcFOS supplementation in infant formula reduce the incidence of atopic dermatitis, allergies, respiratory tract infections, and gastrointestinal infections, including RV infection, during early life [11,12,13,14].

On the other hand, probiotics are defined as live microorganisms, which, when administered in adequate amounts, confer a health benefit to the host [15]. Probiotics can also have a role in immune maturation [16] and prevent and/or treat different diseases, such as gastrointestinal and respiratory infections, colitis, irritable bowel syndrome, and allergies [17].

Postbiotics are described as a preparation of inanimate microorganisms and/or their components that confers a health benefit on the host [15]. The use of postbiotics is a new strategy to obtain the beneficial effects of probiotic bacteria without their possible disadvantages. Even though probiotic-related infections have rarely been reported and the fear that probiotics could express virulence factors and transfer antibiotic resistance genes to pathogenic bacteria [18,19,20] remains controversial, the use of postbiotics could avoid those issues because the bacteria are inactivated by heat, high pressure, sonication, or ionizing irradiation [21]. The mechanism of action of postbiotics is not yet well-known [22], and their efficacy depends on the type of the postbiotic used: microbial metabolites, carbohydrates, organic acids, lipids, proteins, cell wall components, or other fermented products generated in the matrix [17]. Many studies report that postbiotics could improve physiological functions [23], as well as prevent and treat diseases, related to gut barrier dysfunction, gastroenteritis, respiratory tract, and enteric infections [22,24,25,26].

For many years, the literature has focused on different nutritional interventions using prebiotics and probiotics in the context of gastrointestinal infections [1,13,27,28,29]. Recently, different research has showed the beneficial effects of postbiotics on gastrointestinal infections [11,22,24]. The present study aimed to examine the effects of a daily supplementation with a formulation based on a postbiotic and prebiotic mixture, as occurs in breast milk, in a model of RV infection in suckling. The postbiotic is constituted by an inactivated fermented milk infant formula obtained from *Bifidobacterium breve* and *Streptococcus thermophilus* activity by an innovative fermentation process (Lactofidus™). The prebiotic is based on a mixture of scGOS and lcFOS (9:1). The products were administrated separately or in combination, and their actions on growth, immune variables, microbiota composition, and short-chain fatty acid (SCFA) production, among others, were evaluated.

## 2. Materials and Methods

### 2.1. Animals

Fifteen pregnant Lewis rats (G14), purchased from Janvier (Le Genest-St-Isle, France), were individually housed in cages (2184 L Eurostandard Type II L, Tecniplast, West Chester, PA, USA) that contained bedding of large fibrous particles and tissue papers (Souralit 1035, Bobadeb S.L., Santo Domingo de la Calzada, Spain and Gomà-Camps S.A.U., La Riba, Spain, respectively). Rats were monitored daily and allowed to deliver at term. The birth day was registered as day 1 of life. On day 2, litters were randomly assigned to 5 experimental groups and were culled to 8 neonatal rats per mother, with a similar proportion (40–60%) of each sex/litter. Dams were fed with a commercial diet corresponding to the American Institute of Nutrition (AIN) 93 G formulation [30] (Teklad Global Diet 2014, Envigo, Indianapolis, IN, USA) and water ad libitum. Neonatal rats had free access to maternal milk and commercial diet. Animal handling was performed after separating the mother and keeping the suckling rats in the home-cage. Then, oral administration was performed randomly. Animals were housed under controlled conditions of temperature (20–24 °C) and humidity (40–60%) in a 12 h light–12 h dark cycle (lights on at 8 h and lights off at 20 h) in a special safe and isolated room, designed and authorized for working under biosecurity level 2 conditions, at the Animal Service of the Faculty of Pharmacy and Food Science, at the University of Barcelona. Animal procedures were approved by the Ethical Committee for Animal Experimentation of the University of Barcelona and the Catalonia Government (CEEA/Ref. 255//18 and PAMN/Ref. 10176, respectively) in accordance with the EU-Directive 2010/63/EU. The sample size required was calculated by the Appraising Project Office’s program from the Universidad Miguel Hernández de Elche (Alicante). The minimal number of animals to provide statistically significant differences among groups, using the diarrhea score as a variable and assuming that there was no dropout rate and type I error of 0.05 (two-sided), was 3 L per group, as in previous studies, due to the high differences among litters [11,31,32].

### 2.2. Experimental Design

Pups were distributed into 5 groups of 24 animals each (three litters of eight animals/group): the reference (REF) group, rotavirus-infected (RV) group, and three RV-infected groups supplemented with a mixture of scGOS and lcFOS in 9:1 ratio (RV + PRE), Lactofidus^TM^ (RV + POST), and their combination (RV + P/P). Supplementations were obtained from Danone Nutricia Research (Utrecht, The Netherlands). This prebiotic mixture has evidenced, in vitro, in vivo, and in clinical trials, its similarity, in terms of function and structure, to those present in breast milk.

Pups were orally administered on a daily basis by oral gavage, as previously described [27], with the normalized volume/body weight of vehicle, prebiotic, postbiotic, or their combination (9 µL/g/day), from the day 2 of life to the 16 day of life—the end of the strict lactation period. The animals from the RV + PRE group were supplemented with 0.8 g of scGOS/lcFOS per 100 g of body weight. GOS/FOS is a mixture of GOS (Vivinal GOS, Borculo Domo, Zwolle, The Netherlands) with a degree of polymerization (dp) of 3–8, as well as long-chain FOS (Raftiline HP, Orafti, Wijchen, The Netherlands; average dp > 23) in a 9:1 ratio.

The RV + POST group was fed with 0.92 g/100 g body weight of a heat inactivated milk fermented by the bacteria *Bifidobacterium breve* and *Streptococcus thermophilus* (Lactofidus^TM^) [33,34,35,36]. During the fermentation of the milk matrix, both strains were metabolically active and produced bioactive compounds (postbiotics); after that, the milk matrix was spray dried and then used.

The RV + P/P group received both supplements at the same doses as when given separately and maintaining the volume of administration (9 µL/g/day). The REF and RV groups received a matched volume of water. Thus, the REF group constituted the non-infected non-supplemented control group. The selection of the product doses was based on previous works [13,23,27,32,37,38].

The simian SA-11 RV strain was provided by the Virus Enteric group from the University of Barcelona. Viruses were propagated in fetal African green monkey kidney cells (MA-104) and titrated as TCID_50_/mL (TCID, tissue culture infection dose) [39]. On day 5 of life, SA-11 was inoculated by oral gavage (2 × 10^8^ TCID_50_ RV/rat) in 100 µL of phosphate buffered saline (PBS) to suckling rats, as previously described [39], with the exception of REF animals, which received the same volume of PBS. The RV was inoculated after 1 h of separation from their dams to avoid interference between the RV and milk components.

Body weight was recorded each day and clinical evaluation was performed daily from the day 4 of life to the day 16 of life at the same time to disturb the animals just once. The naso–anal and tail lengths were measured to calculate the body/tail ratio the end sampling day (day 8 or day 16). In addition, body mass index (BMI) was calculated as body weight/length^2^ (g/cm^2^) and the Lee Index was calculated as (weight^0.33^/length) × 1000 (g^0.33^/cm). Half of the animals of each litter were euthanized at day 8, to analyze variables associated at the peak of diarrhea and the other half at day 16, to determine the effects of the dietary interventions post-diarrhea.

### 2.3. Clinical Evaluation and Fecal Specimen Collection Design

RV infection was evaluated from day 4 to 16 by the evaluation of the animal weight and clinical indexes derived from fecal samples. After gentle abdominal massage, fecal samples were obtained, scored, and frozen at −20 °C for further analysis. The severity of diarrhea was expressed by fecal weight and by scoring fecal samples from 1 to 4 (diarrhea index [DI]) based on color, texture and amount as described: (1)—normal; (2)—soft yellow-green; (3)—totally loose yellow-green; and (4)—high amount of watery feces. Scores ≥ 2 indicate diarrheic feces, whereas scores < 2 indicate absence of diarrhea [39].

The area under the curve of severity (sAUC) during days 5 to 10 was calculated as a global value of severity. The maximum diarrhea index (MDI)—the highest value during the diarrhea period—was also calculated. Incidence of diarrhea was expressed as the percentage of diarrheic feces (% DF, considering the number of total feces collected daily in each group) and by the percentage of diarrheic animals (% DA, consisting of the percentage of pups presenting diarrhea in each group). The AUC of % DF and % DA (dfAUC and daAUC) from days 5 to 10 were measured as global values of incidence. The maximum percentage of diarrheic feces (MDF) and diarrheic animals (MDA) were defined as the highest scores during the diarrhea period. The days when MDI (MDId), MDF (MDFd), and MDA (MDAd) were achieved were also used as indicators. Finally, the interval between the day of diarrhea beginning (DDB) and the day of diarrhea ending (DDE) was measured to calculate the diarrhea period (DP) for each animal. The number of days with diarrhea within the diarrhea period were calculated (days with diarrhea, DwD).

The DI, MDI, sAUC, % DF, % DA, dfAUC, daAUC, MDF, MDA, DP, and DwD were normalized in RV + PRE and RV + P/P groups due to intrinsic fecal aspects of scGOS/lcFOS intervention, as previously described [27]. The data from REF, RV, and RV + POST were not normalized, due to no basal effect being observed. The data normalization was calculated by subtracting the mean DI of the timepoints when there was no diarrhea in the RV group. The difference between this mean and the baseline score (DI = 1) was subtracted to all values of DI. To ascertain the effects of scGOS/lcFOS and Lactofidus™-scGOS/lcFOS on RV infection, the normalized data reflects better their effect.

On day eight and sixteen, half of the pups/dam each day were intramuscularly anesthetized with ketamine (90 mg/kg) (Merial Laboratories S.A., Barcelona, Spain) and xylazine (10 mg/kg) (Bayer A.G., Leverkusen, Germany). On those days, morphometric variables such as the weight of stomach, small and large intestines, liver, thymus and spleen were obtained. Moreover, small and large intestines’ length was recorded. A 1 cm central portion of small intestine was conserved in RNAlater^®^ (Ambion, Applied Biosystems, Austin, TX, USA), incubated at 4 °C overnight and frozen at −20 °C until PCR analysis. To obtain gut wash, the remaining parts of the intestine were opened lengthwise, cut into 5 mm pieces, incubated with 2 mL of PBS in a shaker (10 min, 37 °C) and centrifugated. The supernatants were frozen at −20 °C until alpha-1 antitrypsin (A1AT) analysis. To measure the immunoglobulin (Ig) pattern and anti-RV antibodies (Ab), plasma samples were used. Furthermore, stomach content was obtained to measure the pH and to study the anti-RV Ab levels. Moreover, colorectal content was used to study the microbiota composition (day 8). Finally, SCFAs were measured in the cecal content, at day 16.

### 2.4. Stool and Stomach Content pH

Fecal samples from the peak of diarrhea (days 7–9) and from the end of the study (days 14–16) were diluted in distilled water (up to 200 mg/mL) and agitated, whereas stomach content samples from day eight and sixteen were measured directly without previous dilution. The measure of pH was performed using a 5207 pH electrode for surfaces and a micropH 2001 pH meter (Crison Instruments, Barcelona, Spain).

### 2.5. Fecal SA-11 Shedding

Feces from day 6 were diluted in PBS (10 mg/mL), homogenized using Pellet Pestles Cordless Motor (Sigma-Aldrich, Madrid, Spain), and centrifuged (170 g, 5 min, 4 °C). Supernatants were stored at −20 °C until analysis. SA-11 virus particles were measured by ELISA as previously described [11].

### 2.6. In Vitro Blocking Assay

To test the ability of scGOS-lcFOS and the Lactofidus^TM^ to bind the SA-11 particles, an in-house in vitro blocking assay was performed [13,27]. RV was diluted with 1% PBS-Tween to reach the concentration of 5 × 10^4^/mL—the highest concentration previously observed in stools of RV-infected suckling rats [13]. Starting from the concentration administered to neonatal rats, different dilutions (from 1/2 to 1/32) of Lactofidus™ and/or scGOS/lcFOS were preincubated with the virus at 1/1 ratio for 30 min. Then, free, noncoated viral particles were quantified by ELISA, as described above (fecal SA-11 shedding).

### 2.7. Intestinal Permeability Assay

The rat SERPINA1/Alpha 1 Antitrypsin ELISA kit (LifeSpan Biosciences Inc., Seattle, WA, USA) was used to measure the levels of A1AT in the gut wash—a marker of intestinal permeability—as in previous works [13]. The standard concentrations ranged from 100 to 1.563 ng/mL. The assay sensitivity was 1.56 ng/mL.

### 2.8. Anti-RV Antibodies

Anti-RV IgM and anti-RV total Ig in neonatal rats’ plasma were measured by ELISA, following previous procedures [27,39]. In addition, the frozen stomach content from 16-day-old rats was homogenized, diluted in PBS, and tested to quantify anti-RV Ab.

### 2.9. Quantification of Immunoglobulins

At day 16, plasma concentration of IgG isotypes, IgA and IgM was measured using ProcartaPlex™ Multiplex immunoassay (Thermo Fisher Scientific, Barcelona, Spain), as described in previous studies [13,23]. The specific concentration of each Ig was measured by MAGPIX^®^ analyzer (Luminex Corporation, Austin, TX, USA) at the Scientific and Technological Centers of the University of Barcelona (CCiT-UB).

### 2.10. Gene Expression Analysis

On day 16, a 1 cm central portion of the small intestine was homogenized, as previously described [38]. RNA was isolated with the RNeasy^®^ Mini Kit (Qiagen, Madrid, Spain), and its purity and concentration were determined with a NanoPhotometer (BioNova Scientific S.L., Fremont, CA, USA). Afterwards, a thermal cycler PTC-100 Programmable Thermal Controller and TaqMan^®^ Reverse Transcription Reagents (Applied Biosystems, AB, Weiterstadt, Germany) were used to obtain the corresponding cDNA.

The real-time PCR was performed using the ABI Prism 7900 HT (AB) with the specific PCR TaqMan^®^ primers (AB): Blimp-1, FcRn, IgA, Cldn2, Cldn4, Ocln, Muc2, Muc3, TLR 2, TLR3, TLR4, TLR5, TLR7, and TLR9, normalized by the endogenous gene, Gusb, using the 2-ΔΔCt method. Results are expressed as the percentage of expression in each experimental group, normalized to the mean value obtained for the REF group, which was set at 100%.

### 2.11. Colorectal Microbiota Composition

On day 8, DNA from samples of colorectal content obtained (6 animals/group) were amplified 25 PCR cycles. To ensure quality control, a negative control of the DNA extraction and a positive Mock Community control were included. Later, they were sequenced in the V3–V4 variable region of the 16S rRNA gene. The Illumina Miseq sequencing 300 × 2 approach was assessed (Illumina Inc., San Diego, CA, USA), and sequences were merged and processed using MiSeq run and MiSeq Reporter (on-system software) in collaboration with Microomics (Barcelona, Spain).

The number of observed operational taxonomic units (OTUs, i.e., richness), Pielou’s evenness and Shannon’s diversity indexes were calculated to estimate the alpha biodiversity. Unweighted Unifrac distance was measured to assess the beta diversity. The taxonomic assignment of phylotypes was performed using a Bayesian Classifier, trained with Silva database version 132–99% OTUs full-length sequences [40]. The relative proportions of families and genera were calculated and represented with stacked bars. The category “others” represented in each graph includes those families whose presence was lower than 1% in the REF group and those genera whose presence was lower than 3% in the same group.

Venn diagrams were created to study the presence or absence of taxonomic ranks (family and genera) in the experimental groups. A bacterial group was considered as present when all neonatal rats displayed proportions higher than 0.001%, while the bacterial groups detected in less pups, or in a lower proportion, were regarded as absent.

### 2.12. Quantification of Short-Chain Fatty Acids in the Cecal Content

On day 16, cecal content samples were obtained to measure cecal SCFA (acetic, propionic, butyric, valeric, isobutyric, and isovaleric acids) content by headspace-gas chromatography-mass spectrometry (HS-GC-MS) at the GC-MS unit of the CCiT-UB, as previously described [13,23]. The lower limits of detection (in μmol/g of feces) were 0.404 for acetic acid, 0.068 for propionic acid, 0.020 for butyric acid, 0.001 for valeric acid, 0.003 for isobutyric acid, and 0.001 for isovaleric acid.

### 2.13. Statistical Analysis

Results are expressed as mean ± SEM. For statistical analysis, the Statistical Package for Social Sciences (SPSS v22.0) (IBM, Chicago, IL, USA) was used. Data were tested for normality distribution (by Shapiro–Wilk) and homogeneity of variance (by Levene’s test). A conventional one-way ANOVA test was carried out followed by the post hoc Bonferroni when data had a normal behavior and were homogeneous. The nonparametric Kruskal–Wallis test followed by the post hoc Mann–Whitney U test was performed when data were neither equals nor normally distributed. This last test was also used for non-parametric variables such as the ID score. As sexual dimorphism has shown to have low impact on the response against RV-infected suckling rats [41] and the low sample size when distributing by sex, differential sex-effects were not compared.

Kruskal–Wallis test was assessed to analyze microbiota alpha diversity. To calculate principal coordinates analysis (PCoA) beta diversity distance matrices were used.

To determine the significance of groups present in community structure, R software package version 3.6.0. Permanova and ANOSIM tests were used. A Permdisp test was used to identify location vs. dispersion effects [42]. ANCOM [43] and Kruskal–Wallis test were used to test differential relative abundance of taxa. After the Kruskal–Wallis test, Conover’s test with FDR, Benjamini–Hochberg correction was added for pairwise comparison. Finally, Biodiversity R version 2.11-1, PMCMR version 4.3, RVAide Memoire version 0.9–7, and vegan version 2.5–5 packages were used for the different statistical analysis performed. Significant differences were established when *p* < 0.05.

## 3. Results

### 3.1. Growth and Morphometry

**Figure 1:**
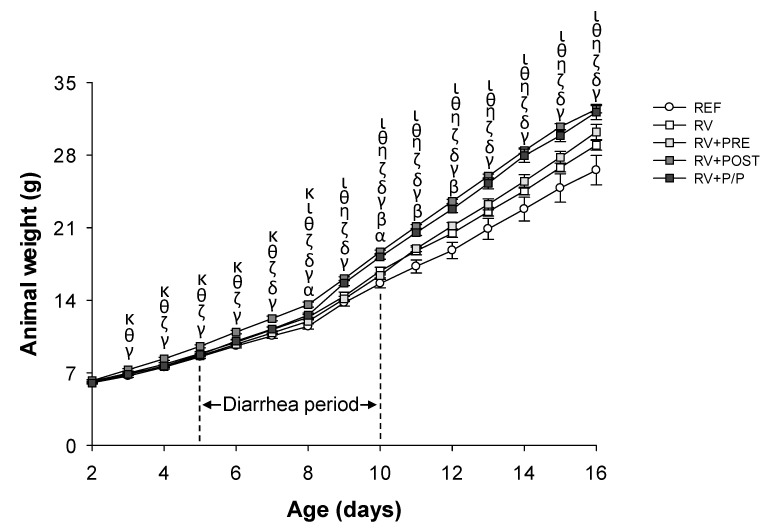
Body weight (g) during the study (day 2 to day 16 of life). Results are expressed as mean ± SEM (*n* = 24 animals/group). Statistical differences: ^α^ *p* < 0.05 RV vs. REF, ^β^ RV + PRE vs. REF, ^γ^ RV + POST vs. REF, ^δ^ RV + P/P vs. REF, ^ζ^ RV + POST vs. RV, ^η^ RV + P/P vs. RV, ^θ^ RV + POST vs. RV + PRE, ^ι^ RV + P/P vs. RV + PRE, and ^κ^ RV + P/P vs. RV + POST. REF: reference group; RV: rotavirus group; RV + PRE: rotavirus group supplemented with a mixture of scGOS/lcFOS; RV + POST: rotavirus group supplemented with Lactofidus^TM^; RV + P/P: rotavirus group supplemented with the combination of both.

The body weight was recorded from day 2 to day 11 of life (Figure 1). The diarrhea of the animals was mild and did not affect their growth, even though the body weight of RV animals was higher compared to the REF group (on day 8 and day 10, *p* < 0.05). In addition, RV + PRE showed an increase in body weight compared to REF animals (on day 10 and day 11). The body weight in RV + POST group was higher compared to REF (from day 3 to day 11), RV (from day 4 to day 11), RV + PRE (from day 3 to day 11), and RV + P/P (between day 3 and day 8). In addition, the body weight of RV + P/P increased compared to REF (from day 7 to day 11), RV (from day 9 to day 11), and RV + PRE (from day 8 to day 11) (*p* < 0.05).

The growth-associated measurements and relative weight of organs were recorded at the peak of diarrhea (day 8) and at the end of the study (day 16). Despite observing no differences in growth-associated measurements, or in the relative weight of organs, between RV and REF groups on day 8 and day 16 (Table 1 and Table S1, respectively), the supplemented groups showed some differences.

As can be observed in Table 1, the mean Lee index of the RV + PRE group was lower than that of the animals in the RV and RV + POST groups (*p* < 0.05). In addition, RV + POST showed an increase in the BMI compared to REF and RV + PRE (*p* < 0.05). The body/tail length ratio of RV + P + P was higher than that of the REF group, whereas the naso–tail measure was lower compared to RV + POST (*p* < 0.05).

With regard to the relative weight of organs, thymus weight was lower in RV + PRE and RV + P/P compared to REF and RV + POST animals (*p* < 0.05). In addition, RV + POST and RV + P/P increased the relative weight of the spleen compared to the REF and RV groups (*p* < 0.05). An increase in the percentage of the relative weight of the small intestine was observed in all supplemented groups compared to the REF group, with a statistical difference between RV + PRE and RV + P/P with respect to RV and RV + POST groups (*p* < 0.05). In addition, the large intestine length of RV + POST was reduced compared to REF, RV, and RV + P/P, whereas RV + PRE only showed a reduction when it was compared to the REF group (*p* < 0.05). The liver’s weight was higher in RV + POST and RV + P/P compared to RV + PRE (*p* < 0.05). Finally, the small intestine length was decreased in RV + POST compared to RV + PRE and RV + P/P animals (*p* < 0.05) (Table 1).

On day 16 (Supplementary Table S1), RV + POST and RV + P/P groups showed an increase in the naso–anal length compared to REF and RV animals (*p* < 0.05). Moreover, RV + POST showed higher naso–tail length compared to the REF and RV groups (*p* < 0.05). The BMI was increased in RV + POST and RV + P/P animals compared to RV ones (*p* < 0.05). Similarly to day 8, on day 16 the relative spleen weight was higher in RV + POST and RV + P/P group compared to REF animals. RV + POST was the group with the highest relative thymus weight (*p* < 0.05). The relative weight of large intestine of RV + POST was higher than that from the REF group, whereas their length was reduced (*p* < 0.05). RV + PRE and RV + P/P showed an increase in the weight of large and small intestines compared to other groups (*p* < 0.05). Finally, the length of the large intestine was lower in RV + P/P compared to REF and RV animals (*p* < 0.05) (Supplementary Table S1).

With regard to the hematological variables, on day 8 (Supplementary Table S2), RV showed higher mean platelet volume compared to REF and RV + PRE animals (*p* < 0.05). The levels of hemoglobin, mean cell hemoglobin, and platelet counts were higher in RV + PRE group compared to RV (*p* < 0.05). The RV + P/P group showed the highest values of white blood cells, lymphocytes, and mean red cell volume (*p* < 0.05). Moreover, hemoglobin and mean cell hemoglobin were increased compared to REF, RV, and RV + POST animals (*p* < 0.05). Furthermore, neutrophil, monocyte, eosinophil, and basophil counts were higher compared to the REF and RV groups (*p* < 0.05). The levels of monocytes, eosinophils, basophils, blasts, and other precursors of white cells in RV + P/P were present in higher abundance, whereas their percentage was reduced compared to RV (*p* < 0.05). No differences were observed between RV + POST and REF or RV animals (Supplementary Table S2). On day 16 (Supplementary Table S3), the mean red cell volume in the RV + POST and RV + P/P groups was reduced compared to that in the REF group (*p* < 0.05). The hematocrit of RV + P/P group was lower than that in REF animals (*p* < 0.05). No differences were observed among REF, RV, and RV + PRE.

On the other hand, the fecal and stomach content pH were measured during the diarrheic process (on days 7–9 in feces and day 8 in stomach content) and after the diarrhea period (on days 14–16 in feces and day 16 in stomach content) (Supplementary Figure S1). RV + POST showed a lower fecal pH, compared to REF and RV groups (*p* < 0.05), during the diarrhea period (Supplementary Figure S1a). No changes were observed in fecal pH in the post-diarrhea period. If we focus on the stomach pH (Supplementary Figure S1b), all supplemented groups showed a decrease in stomach content pH during the diarrhea period compared to REF and RV animals (*p* < 0.05). No differences were observed during the post-diarrhea period among groups.

### 3.2. Incidence and Duration of the Diarrhea Process

**Figure 2:**
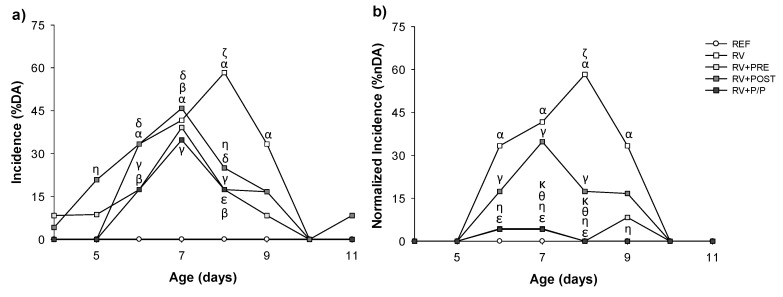
Incidence of RV infection expressed as percentage of diarrheic animals (% DA) by two approaches: non-normalized results (**a**) and normalized results (**b**) from day 4 to day 11 (*n* = 24 animals/group). Statistical significance: ^α^ *p* < 0.05 RV vs. REF, ^β^ RV + PRE vs. REF, ^γ^ RV + POST vs. REF, ^δ^ RV + P/P vs. REF, ^ε^ RV + PRE vs. RV, ^ζ^ RV + POST vs. RV, ^η^ RV + P/P vs. RV, ^θ^ RV + POST vs. RV + PRE, and ^κ^ RV + P/P vs. RV + POST. REF: reference group; RV: rotavirus group; RV + PRE: rotavirus group supplemented with a mixture of scGOS/lcFOS; RV + POST: rotavirus group supplemented with Lactofidus^TM^; RV + P/P: rotavirus group supplemented with the combination of both.

The incidence of RV-induced diarrhea was evaluated from day 4 to 11. As can be observed in Figure 2, the incidence, expressed as the percentage of animals displaying diarrhea (% DA) in the RV group was 33.33% on day 6, the following day it increased to 41.67%, and on day 8, it reached the maximum proportion (58.33%). Afterwards, on day 9, the % DA decreased to 33%, and on day 10, none of the animals in the RV group showed diarrhea. In contrast, the three diets tested were able to reduce the proportion of infected animals. Specifically, all supplemented groups displayed lower % DA (*p* < 0.05) at day 8, when less than 25% of the animals displayed symptoms (*p* < 0.05) (Figure 2a).

After the normalization of data from RV + PRE and RV + P/P groups, the % DA in these groups was even lower, up to 4% (on days 6 and 7), whereas on day 8, none of the animals showed diarrhea (*p* < 0.05) (Figure 2b).

All the supplemented groups tended to reduce other measures of incidence (e.g., maximum percentage of diarrheic animals, MDA) or the overall pattern (e.g., AUC of the incidence curve), especially the POST group (Table 2). The day of the maximum incidence of diarrhea (MDAd) was on day 8 in RV animals, whereas it was one day before (day 7) in the supplemented groups.

If we focus on the duration of the diarrhea, the clinical symptoms started around day 6–7 in all groups and ended 2 days later (Table 2). The two measures of the duration of the diarrhea, the diarrhea period (DP) and the days with diarrhea (DwD), were clearly reduced by the intervention with the POST, as well as in the RV-PRE and RV-P/P, if the normalized results are taken into account (*p* < 0.05, Supplementary Table S4).

### 3.3. Severity of Diarrhea

**Figure 3:**
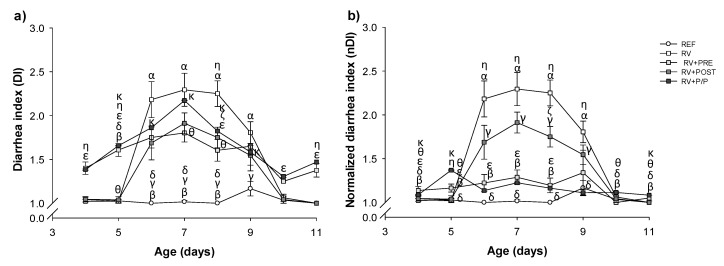
Severity of diarrhea from day 4 to day 11 by two approaches: non-normalized results (**a**) and normalized results (**b**). Results are expressed as mean ± SEM (*n* = 24 animals/group). Statistical significance: ^α^ *p* < 0.05 RV vs. REF, ^β^ RV + PRE vs. REF, ^γ^ RV + POST vs. REF, ^δ^ RV + P/P vs. REF, ^ε^ RV + PRE vs. RV, ^ζ^ RV + POST vs. RV, ^η^ RV + P/P vs. RV, ^θ^ RV + POST vs. RV + PRE, ^κ^ RV + P/P vs. RV + POST, and RV + POST vs. RV + P/P. REF: reference group; RV: rotavirus group; RV + PRE: rotavirus group supplemented with a mixture of scGOS/lcFOS; RV + POST: rotavirus group supplemented with Lactofidus^TM^; RV + P/P: rotavirus group supplemented with the combination of both.

**Figure 4:**
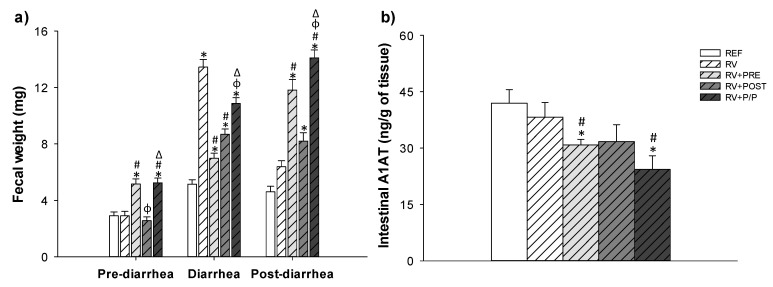
Fecal weight (mg) (**a**) and intestinal barrier permeability (**b**). The fecal weight was grouped in the pre-diarrhea, diarrhea, and post-diarrhea periods (**a**). The pre-diarrhea lasts from day 0 to day 5, diarrhea from day 6 to day 9, and post-diarrhea period from day 10 to day 16. Results are expressed as mean ± SEM (*n* = 24 animals/group). Statistical differences: * vs. REF, ^#^ vs. RV, ^ϕ^ vs. RV + PRE, ^Δ^ vs. RV + POST. REF: reference group; RV: rotavirus group; RV + PRE: rotavirus group supplemented with a mixture of scGOS/lcFOS; RV + POST: rotavirus group supplemented with Lactofidus^TM^; RV + P/P: rotavirus group supplemented with the combination of both.

The severity of the diarrheic process was evaluated from day 4 to day 11 by two approaches: the diarrheic index (DI) and the fecal weight (Figure 3 and Figure 4a). As can be observed in Figure 3a, all supplemented groups showed an overall reduction in the diarrhea severity compared to RV animals, which was significantly different on day 8 (*p* < 0.05). After the normalization of data (Figure 3b), the DI was reduced, in RV + PRE, from day 6 to 8 and from day 6 to 9 in the RV + P/P group (*p* < 0.05).

The sAUC was measured from day 5 to 10, the last day with some animals displaying symptoms (Table 2). The RV animals showed a higher sAUC compared to RV + PRE and RV + POST (*p* < 0.05). The maximum diarrhea index (MDI, Table 2) in the RV + POST supplemented group was the lowest and significantly different to that from the RV group, whereas the day the maximum score was achieved (MDId) was similar among groups. After the normalization of data (Supplementary Table S4), RV + PRE and RV + P/P also showed a reduction in MDI and sAUC compared to RV (*p* < 0.05).

Fecal weight is an objective severity variable of RV-induced diarrhea, and it was also measured as an indicator of severity (Figure 4a). Before the induction of RV infection, RV + PRE and RV + P/P showed higher fecal weight compared to REF and RV animals, due to the prebiotic properties that reduce the fecal consistency, increasing the number of soft feces. However, during the diarrhea period, RV + PRE and RV + POST groups showed lower fecal weight compared to RV animals, indicating that the prebiotics and the postbiotic reduce the severity of diarrhea (*p* < 0.05). In the post-diarrhea period, RV + PRE and RV + P/P showed higher fecal weight compared to REF and RV animals, whereas RV + POST group showed an increment compared to the REF group (*p* < 0.05).

### 3.4. Intestinal Permeability

With respect to the intestinal permeability assay (Figure 4b) performed during the peak of the diarrheic process (day 8), the A1AT levels in the gut of RV animals was similar to the REF. However, RV + PRE and RV + P/P groups had a lower A1AT concentration compared to both REF and RV groups (*p* < 0.05). No changes were observed in the RV + POST group.

### 3.5. Fecal SA-11 Shedding and Blocking Assay

**Figure 5:**
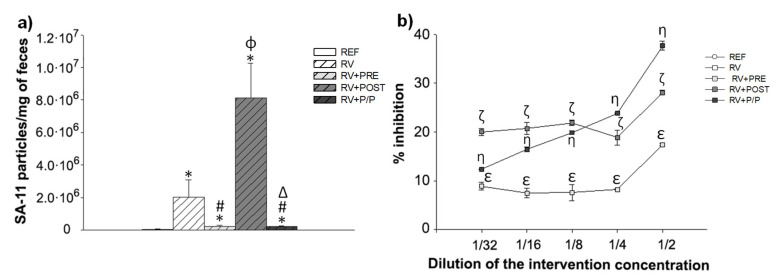
Viral shedding in feces (**a**) and blocking activity of the supplements, which was tested by an in-house in vitro blocking assay (**b**). Results are expressed as mean ± SEM (*n* = 24 animals/group). Statistical differences: * vs. REF, ^#^ vs. RV, ^ϕ^ vs. RV + PRE, ^Δ^ vs. RV + POST (**a**) and ^ε^ RV + PRE vs. RV, ^ζ^ RV + POST vs. RV, and ^η^ RV + P/P vs. RV (**b**). REF: reference group; RV: rotavirus group; RV + PRE: rotavirus group supplemented with a mixture of scGOS/lcFOS; RV + POST: rotavirus group supplemented with Lactofidus^TM^; RV + P/P: rotavirus group supplemented with the combination of both.

The viral shedding in feces (Figure 5a) was determined by ELISA at day 8, which corresponded to the day of maximum elimination of the virus [11]. As expected, an increase in SA-11 particles in feces was observed in the RV group. This increase was drastically reduced in RV + PRE and RV + P/P groups (*p* < 0.05). In contrast, although not statistically different, a tendency to increase the concentration of viral particles was detected in RV + POST, with respect to the RV group (*p* = 0.06).

To assess the binding activity of scGOS/lcFOS and Lactofidus^TM^, an in-house in vitro blocking assay was performed (Figure 5b). The highest percentage of inhibition of the RV + PRE, RV + POST, and RV + P/P was 17%, 28%, and 38%, respectively, at the ½ dilution of the dose used in the in vivo study. Moreover, the combination of the prebiotic and the postbiotic showed a dose-dependent inhibition of RV detection.

### 3.6. Antibody Production

**Figure 6:**
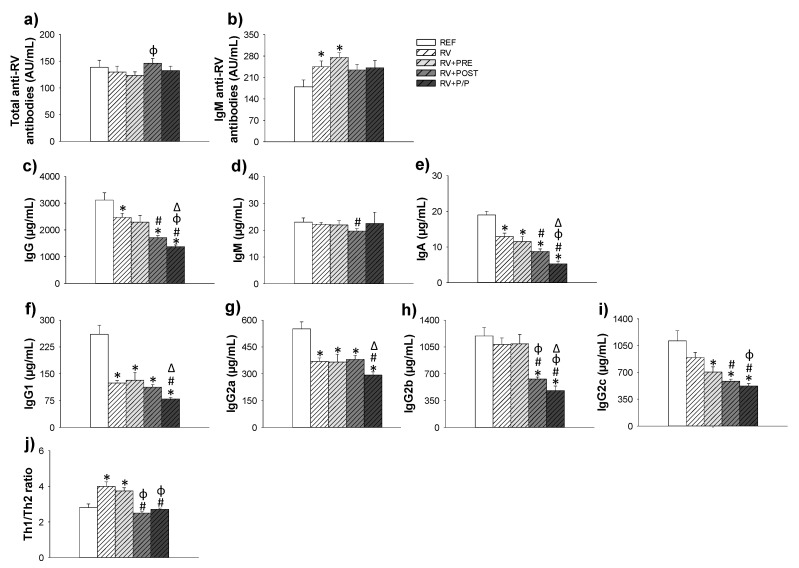
Concentration of total anti-RV antibodies (**a**), IgM anti-RV (**b**), and immunoglobulins in plasma (**c**–**i**) at the end of the study (day 16). Th1/Th2 ratio refers to the relationship between IgG2b + IgG2c and IgG1 + IgG2a (**j**). Results are expressed as mean ± SEM (*n* = 12). Statistical differences: * vs. REF, ^#^ vs. RV, ^ϕ^ vs. RV + PRE, ^Δ^ vs. RV + POST. REF: reference group; RV: rotavirus group; RV + PRE: rotavirus group supplemented with a mixture of scGOS/lcFOS; RV + POST: rotavirus group supplemented with Lactofidus^TM^; RV + P/P: rotavirus group supplemented with the combination of both.

The quantity of total anti-RV and IgM-anti RV antibodies, in the plasma of suckling rats, was measured at the end of the study (day 16). As can be observed in Figure 6a, the total anti-RV antibody levels were not modified due to the RV infection nor the supplementations, although a tendency to increase the levels of total anti-RV antibodies was found in the RV + POST group compared to RV animals (*p* = 0.09). With regard to the IgM anti-RV antibodies (Figure 6b), RV and RV + PRE groups showed higher levels than REF animals (*p* < 0.05), whereas there was only a tendency to increase the levels in the RV + POST and RV + P/P groups compared to REF animals (*p* = 0.06 in both cases). No differences were found between supplemented groups and RV animals.

The RV, as well as the supplementations, induced some changes in the Ig levels, and only the levels of IgM were the least affected. A reduction in the plasma levels of IgA, IgG1 (Th2-associated isotype) and IgG2a (Th2-associated isotype) was observed in all groups, with respect to the REF group, and a reduction in IgG2c (Th1-associated isotype) was observed in all supplemented groups compared to REF ones, and IgG2b (Th1-associated isotype) was reduced in RV + POST and RV + P/P compared to REF group (*p* < 0.05). Therefore, the Th1/Th2 ratio increased in the RV and RV + PRE groups, with respect to REF animals. This increase was not observed in the RV + POST and the RV + P/P, which were able to maintain the REF values.

### 3.7. Gene Expression Analysis

**Figure 7:**
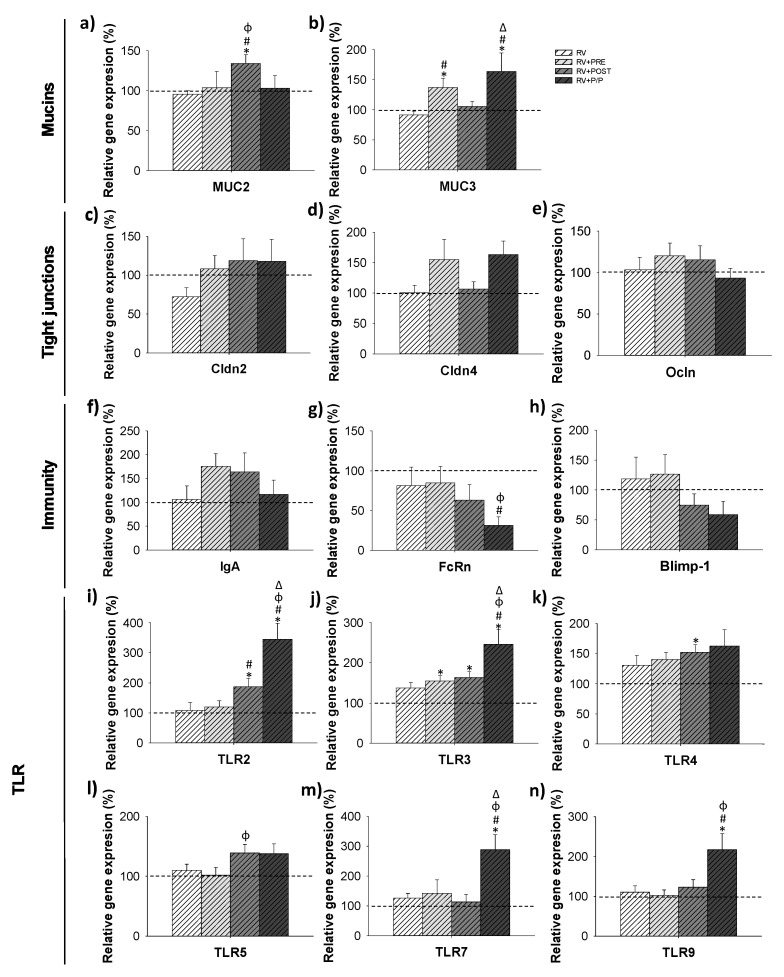
Relative gene expression of mucin (MUC) 2 (**a**) and MUC 3 (**b**), TJ proteins claudin (Cldn) 2, Cldn4 (**c**), and (**d**), respectively, and occludin, (Ocln) (**e**), immunity related molecules IgA (**f**), FcRn (**g**) and Blimp-1 (**h**) and Toll-like receptors (TLR) 2 (**i**), TLR3 (**j**), TLR4 (**k**), TLR5 (**l**), TLR7 (**m**), and TLR9 (**n**) was quantified by real-time PCR on day 16. Relative gene expression was calculated with respect to REF animals, which corresponded to 100% of transcription. Results are expressed as mean ± S.E.M. (*n* = 9 animals/group). Statistical differences: * vs. REF, ^#^ vs. RV, ^ϕ^ vs. RV + PRE, ^Δ^ vs. RV + POST. REF: reference group; RV: rotavirus group; RV + PRE: rotavirus group supplemented with a mixture of scGOS/lcFOS; RV + POST: rotavirus group supplemented with Lactofidus^TM^; RV + P/P: rotavirus group supplemented with the combination of both.

To further study the effect of the different supplementations on the intestinal function, including intestinal barrier, the cross-talk with the microbiota, IgA production, and intestinal maturation, the gene expression of different genes on day 16 was measured in the small intestine (Figure 7). The RV did not induce any change in all the genes assayed; however, the supplementations modified the expression of some of them. With regard to the mucins evaluated, only the RV + POST group showed an increase in the relative gene expression of MUC2 (*p* < 0.05). In contrast, MUC3 showed higher values in the RV + PRE and RV + P/P groups compared to the REF and RV groups (*p* < 0.05). No differences were observed in the relative gene expression of tight junction (TJ) proteins Cldn2, Cldn4, and Ocln.

No differences were observed in the relative expression of IgA, although a tendency to increase the levels was found in the RV + PRE group compared to REF and RV animals (*p* = 0.08 and *p* = 0.07, respectively). With regard to the relative gene expression of intestinal maturation molecules (FcRn and Blimp-1), only a reduction in FcRn was observed in the RV + P/P group compared to RV animals. No differences were observed in the relative expression of IgA and Blimp-1 among groups, although a tendency to increase the levels of IgA was found in the RV + PRE group compared to REF and RV animals (*p* = 0.08 and *p* = 0.07, respectively).

Finally, some changes in the relative gene expression of TLRs were observed, mainly in the RV + P/P group. An increase in RV + POST and RV + P/P was observed in TRL2 compared to REF (×1.9 and 3.5 times, respectively; *p* < 0.05) and RV (×1.7 and ×3.2 times, respectively; *p* < 0.05) animals. Higher expression levels of TLR3 were found in RV + PRE, RV + POST and RV + P/P compared to the REF group (×1.5, ×1.6, ×2.5 times, respectively; *p* < 0.05) and also were higher comparing RV + P/P to RV, RV + PRE and RV + POST animals (*p* < 0.05). The RV + POST group showed an increase in the relative gene expression of TLR4 compared to the REF group (×1.6 times; *p* < 0.05), whereas a tendency to increase was observed in RV + PRE and RV + P/P compared to REF animals (*p* = 0.07 and *p* = 0.08, respectively). Although no changes were observed in TLR5′s expression compared to the REF and RV groups, RV + POST showed higher expression levels compared to RV + PRE (*p* < 0.05) and tended to increase compared to REF levels (*p* = 0.06). The RV + P/P was the only group that showed an increase in TLR 7 and TLR 9 (×2.9 and ×2.2 times, respectively compared to REF group, and ×2.3 and ×2 times, respectively compared to RV animals; *p* < 0.05).

### 3.8. Colorectal Microbiota Composition

**Figure 8:**
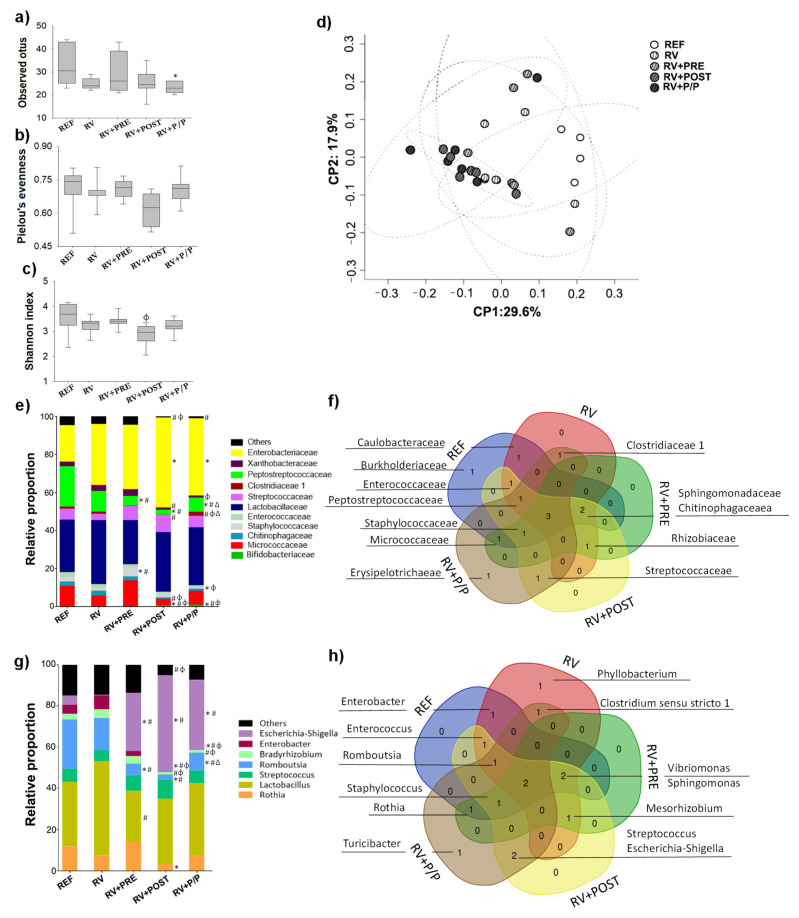
Assessment of fecal microbiota composition on day 8. The sequencing of the amplicon, targeting the V3–V4 variable region of the 16S rRNA, was performed using the Illumina Miseq sequencing 300 × 2 approach. The alpha diversity was represented by the richness (**a**), Pielou’s evenness (**b**), and Shannon’s indexes (**c**). The beta diversity was calculated, measuring the Unweighted Unifrac distance (**d**). The main taxonomic group abundances, corresponding to family (**e**) and genera (**g**) were represented in stacked bars. The qualitative assessment of microbiota was represented in a Venn diagram at the level of family (**f**) and genera (**h**). In this last approach, a bacterial group was considered as present when all six animals displayed proportions higher than 0.001%, while the bacterial groups detected in less animals, or in lower proportion, were regarded as absent. Results derived from *n* = 6 animals/group. Statistical differences: * vs. REF, ^#^ vs. RV, ^ϕ^ vs. RV + PRE, ^Δ^ vs. RV + POST. REF: reference group; RV: rotavirus group; RV + PRE: rotavirus group supplemented with a mixture of scGOS/lcFOS; RV + POST: rotavirus group supplemented with Lactofidus^TM^; RV + P/P: rotavirus group supplemented with the combination of both.

The colorectal microbiota composition was analyzed on day 8 (Figure 8). The alpha diversity of microbial populations was measured by the richness, Pielou’s evenness and Shannon’s indexes (Figure 8a–c, respectively). As can be observed in Figure 8a, the RV + P/P group showed a reduction in richness compared to REF animals. Moreover, the Shannon index of RV + POST was lower than that of RV + PRE animals (Figure 8c). No changes were observed in Pielou’s evenness between groups. Beta diversity was calculated, measuring the Unweighted Unifrac distance (Figure 8d). In this analysis, rats from the REF group had a tendency to form a cluster apart from the other animals (RV, RV + PRE, RV + POST, and RV + P/P groups) who showed similar beta diversity distances.

To further characterize the microbiota a presence/absence analysis of bacterial groups after the different supplementations was performed and visualized with Venn diagrams. This presence/absence analysis was restrictive, and only when a bacterial family was detected in all the animals of a group, this bacterial family was considered as present in the group (Figure 8f). Only three families were present using this approach in all groups, thus being in the core of the Venn diagram: *Enterobacteriaceae*, *Lactobacillaceae*, and *Xanthobacteraceae*. Only in the RV + P/P group was the family *Erypelotritrichaeae* considered to be present. All supplemented groups and REF, but not RV animals, showed the presence of the *Staphylococcaceae* family. In addition, *Micrococcaceae* was found in REF, RV + PRE, and RV + P/P. *Rhizobiaceae* was observed in RV, RV + PRE, and RV + POST, whereas *Clostridiaceae 1* was found in RV and RV + P/P groups. *Streptococcaceae* was present only in RV + POST and RV + P/P. Finally, *Sphingomonadaceae* and *Chitinophagaceae* were not found in RV + P/P.

As we observed at family level, no differences were found between REF and RV animals in genus level. However, supplemented groups showed different relative proportions of bacteria (Figure 8g). The relative proportion of *Escherichia*-*Shigella* increased in all supplemented groups, whereas *Romboutsia* decreased in comparison to REF and RV animals (*p* < 0.05). *Lactobacillus* levels decreased in RV + PRE compared to RV group (*p* < 0.05). RV + POST group showed reduced levels of *Rothia* compared to REF animals (*p* < 0.05). *Enterobacter* proportion decreased in RV + POST and RV + P/P compared to REF and RV groups (*p* < 0.05). Finally, a reduction in *Bradyrhizobium* was found in RV + POST and RV + P/P compared to RV group (*p* < 0.05). In addition, many minority genera were also affected by the infection and by the nutritional interventions (Supplementary Table S5).

With regard to the presence/absence analysis performed by Venn diagrams, only two genera (*Lactobacillus* and *Bradyrhizobium*) were present in the core of the Venn diagram using this restrictive approach. RV + P/P group had the exclusive presence of the genera *Turicibacter*. In addition, *Streptococcus* and *Escherichia*-*Shigella* were found only in RV + POST and RV + P/P. *Rothia* was found in REF, RV + PRE, and RV + P/P. As we observed at family level, all supplemented groups and REF rats showed the *Staphylococcus* genre (Figure 8h).

### 3.9. Quantification of Short-Chain Fatty Acids

The amount of total SCFA and acetic, propionic, isobutyric, butyric, isovaleric, and valeric acid proportion, in the cecum of the suckling rats, was measured on day 16 (Table 3).

The total SCFA levels in the cecum were similar between groups. However, the percentage of acetic acid was higher in RV + PRE and RV + P/P compared to REF and RV animals (*p* < 0.05). On the other hand, the proportion of propionic, isobutyric, and isovaleric were lower in RV + PRE compared to REF and RV, whereas RV + P/P only showed a reduction in propionic acid compared to REF, RV, and RV + POST animals (*p* < 0.05). Moreover, RV + P/P increased the proportion of valeric acid compared to REF and RV + POST animals (*p* < 0.05). No differences were observed in RV and RV + POST compared to the REF group. However, changes in all SCFAs except valeric acid were found between RV + POST and RV + PRE groups (*p* < 0.05).

## 4. Discussion

RV is the main etiological agent of acute gastroenteritis in children. Vaccination, oral rehydration, and dietary interventions are used as preventive and therapeutical treatments to manage gastrointestinal symptoms [1,4]. In the present work, scGOS-lcFOS, Lactofidus^TM^, and their combination have been evaluated on a model of early life RV infection in suckling rats.

Despite the fact that RV infection causes intestinal malabsorption, which is normally associated with fluid loss and dehydration, inducing a reduction in the body weight, in the present study, the moderate diarrhea induced by RV SA-11 strain did not lead to a weight loss as in previous studies [13,27,32,37]. In this regard, supplemented animals showed a higher body weight compared to REF animals, as well as a higher body growth of RV + POST and RV + P/P animals. In previous studies from our group using this model, a nutritional intervention with scGOS-lcFOS slightly increased [13], or did not have an impact on, body weight [11,27]. Moreover, other researchers did not find changes in body weight and growth in pre-weaning rats supplemented with scGOS-Inulin [37]. The impact of Lactofidus^TM^ on growth has also been studied in healthy babies [8]. In infants fed with infant formula with Lactofidus^TM^ and oligosaccharides, a positive effect on height with respect to each product alone has been found [44]. On the other hand, in the present study, a trophic effect on the small and large intestine was observed in all supplemented groups, along with an increase in spleen (RV + POST and RV + P/P animals) and thymus (RV + POST group) weight. A previous study from our group also observed that fermented milk, combined with scGOS-lcFOS, had a trophic effect on the intestines of suckling rats [37]. In accordance with our work, F. Indrio et al. observed that fermented formula in healthy infants increased thymus size compared to infants fed with standard infant formula [45]. The increase in thymus size, as well as that of the spleen, could be linked to a better immune development and response to infection [46].

With regard to clinical signs, all supplemented groups showed a reduction in the incidence and severity of diarrhea, analyzed either by the score or the fecal weight obtained. The prebiotic mixture alone induced softened stools, thus masking its effect against the RV; therefore, results were normalized, as previously performed [27]. In addition, all the supplemented groups showed a reduction in the duration of diarrhea, suggesting the postbiotic, the prebiotic mixture, and the combination as useful tools in the diarrhea prevention repertoire. These positive results agree with previous studies, in which we also observed these changes after scGOS/lcFOS supplementation [13,27]. In addition, in one study, infants that were fed with infant formula fermented with *Bifidobacterium breve* c50 and *Streptococcus thermophilus* 065 also showed a reduction in the severity of diarrhea [47]. Besides the effect, the mechanisms of action of the interventions were evaluated.

On the one hand, we found that the POST reduced fecal pH of infected animals and that all interventions induce a reduction in the pH of the stomach content. Very few data are found in the literature regarding the effect of these compounds in this last compartment, either in animals or in humans. This result may suggest a positive effect on the prevention of pathogens’ entry into the gastrointestinal tract, similar to that of the lower fecal pH [48,49] and could explain the prevention of diarrhea in the supplemented groups.

Another possible mechanism involved in the amelioration of diarrhea could be the improvement in the intestinal barrier function, conferring higher resistance to infection. It is well known that plasma levels of A1AT are elevated during the inflammatory response and can be transported to the intestinal lumen due to the increased permeability of the intestinal epithelial barrier [50]. Therefore, the lower levels of A1AT, found in RV + PRE and RV + P/P groups, could be due to an enhanced barrier effect, thus protecting them from RV infection. In this regard, a previous study from our group also observed a decrease in the A1AT levels in suckling rats supplemented with scGOS/lcFOS and 2′-fucosyllactose [13]. Moreover, other researchers have demonstrated an improvement in the intestinal barrier in obese adults treated with scGOS [51].

The viral shedding reflects the viral particles produced in the intestine. RV + PRE and RV + P/P groups showed a drastic reduction in fecal SA-11 particles, which could be due to the scGOS/lcFOS ability to bind to the virus, as it has been previously reported as an important protection mechanism during RV infection [11,13]. Therefore, scGOS/lcFOS could partially block the pathogen, reducing the ability of the RV to infect the host, consequently reducing the severity of diarrhea [11,27]. In contrast, postbiotic supplementation did not reduce the viral shedding; however, in vitro, it was able to partially block the RV, suggesting that the main mechanism of action involved in the amelioration of the diarrhea is different from that of scGOS/lcFOS.

The titer of total anti-RV antibodies at this early age was not found to be increased after RV infection. As we previously reported [13], there is not a clear specific anti-RV Ig response to the virus due to the immaturity of the immune system of suckling rats. However, in the present study we only found that IgM anti-RV plasma levels increased in RV + PRE compared to the REF group. In previous work by our group, we also found this increase in scGOS/lcFOS supplemented rats [27]; however, in another study, we did not observe this [13]. The higher IgM anti-RV levels in this group could suggest that scGOS/lcFOS supplementation is inducing the maturation of immune system.

The Ig profile changed in the supplemented groups, with IgG and IgA levels reduced in all supplemented groups. In line with this, we previously showed a reduction in IgG1 plasma levels of suckling rats supplemented with scGOS/lcFOS [13]. Furthermore, it was reported that fermented milk, combined with scGOS/lcFOS, increased the levels of IgG (on day 14 of life) and reduced the levels of IgG (on day 21) and IgA (on day 14) [37]. However, the most interesting issue is the overall control by the products of the RV-induced shift to Th1 response, especially those with the postbiotic. In this sense, the Th1/Th2 ratio associated Ig (Th1/Th2 ratio refers to the relationship between IgG2b + IgG2c and IgG1 + IgG2a), which was raised by the RV infection in an attempt to control the infection and was not found in RV + POST and RV + P/P groups. This may suggest that interventions by reducing the infection were consequently avoiding the activation of the pro-inflammatory Th1 response activated due to the RV. To further study the mechanisms responsible for the amelioration of the diarrhea induced by the supplements, we measured the gene expression of genes involved in the barrier function and maturation of the intestine. In the intestine, goblet cells form gel-forming mucins such as MUC2 and enterocytes express transmembrane MUC3 [52]. Along with other immune components, the mucus and the glycocalix limit the number of intact bacteria that can reach the epithelium. The postbiotic increased the levels of MUC2 whereas scGOS/lcFOS, combined or not with Lactofidus^TM^, induced the expression of MUC3. These effects in the infection context on the expression of MUC genes agree with those found in a healthy model of suckling rats [23]. We also observed that scGOS/lcFOS reduced the levels of MUC2 in 8-day-old suckling rats [13]. However, other studies did not observe those changes in MUC2 and MUC3, after scGOS or lcFOS intervention, either in rats or in mice [53,54]. On the contrary, in an in vitro approach, S. Figueroa-Lozano et al., reported that scGOS containing lactose incremented the expression of MUC2 in LS174T cells [55]. The enhancement of both mucins observed in this study suggests a reinforcement of the intestinal barrier function of the suckling rats that may explain the amelioration of the RV-induced diarrhea. This reinforcement might only be at the mucus level because no differences were observed in TJ proteins such as Cldn2, Cldn4, and Ocln gene expression, as also observed in a healthy environment [23]. TJ proteins present in the basolateral membrane of the enterocytes form a barrier that, among other functions, protect the organisms against the entry of pathogens [56,57]. In agreement with our results, Alizadeh et al. did not observe changes in Cldn1-4 in piglets supplemented with scGOS. However, they did observe an increase in the expression of Ocln [58]. In contrast, a previous study from our group showed an increase in Cldn2 in rats infected with SA-11 and supplemented with scGOS/lcFOS [13].

The expression of genes related to immune maturation and regulation (IgA, FcRn, and Blimp-1) were also studied. The levels of FcRn were reduced in the RV + P/P group, indicating an increase in the maturation of the intestine of these animals, as it has been reported that lower levels of FcRn correlate with a higher level of intestinal epithelial barrier maturation [59]. In this study, the postbiotic or scGOS/lcFOS alone did not modify the expression of these genes, in line with that found in a recent study in healthy suckling rats [23]. TLRs are pattern recognition receptors that play a role in the induction of innate immune and inflammatory responses [60]. An increase in TLR gene expression was observed, and some differences regarding the induced changes in noninfected vs. infected rats appeared [23]. In the infection scenario, the combination—but not the products alone—was able to increase the expression of TLR7 and TLR9 genes. This effect could suggest that the combination of scGOS/lcFOS and Lactofidus^TM^ is more effective in enhancing the immune system to fight against infections than the prebiotics or the postbiotics alone.

Gut microbiota composition was also evaluated to study the effects of prebiotic and/or postbiotic supplements in the RV-induced animal model. The alpha diversity of the microbial population showed that RV + P/P decreased the number of observed OTUs compared to those of the REF group. Thus, although a great impact of the infection or the products was not observed, again, the combination is more effective than the products alone, which is in line with a previous study [23]. In the present study, changes in microbial composition were found due to interventions. *Bifidobacteriaceae*, suggesting the prebiotic effect of these compounds, and *Enterobacteriaceae* families increased in RV + POST and RV + P/P. If we focus on the genus level of these bacteria families, *Escherichia*-*Shigella* was increased in all supplemented animals. These groups are usually present in infant feces, but their role in early life is not well established. It is difficult, then, to conclude the impact of the change observed due to the postbiotic intervention. However, a reduction in *Escherichia*-*Shigella* abundance in diarrheal neonatal piglets with respect to their healthy siblings has been reported [61]. Thus, the increase in *Escherichia*-*Shigella* in all supplemented groups makes us consider that this genus could promote some positive effect during the RV infection. This effect will need further attention in future studies.

With regard to SCFA production, none of the supplementations modified the total amount of SCFAs. However, some changes were observed in their proportion. In agreement with our results, the presence of scGOS/lcFOS in an infant formula changed the SCFA pattern, increasing the proportion of acetate and reducing the proportion of propionate and other SCFAs [44]. Furthermore, infants fed with a fermented infant formula with *Bifidobacterium breve C50* and *Streptococcus thermophilus O65* and/or the prebiotics scGOS/lcFOS had lower isovaleric acid levels, although they also found reduced levels in butyric acid in the fermented infant formula with the postbiotics and prebiotics [62]. Previous work by our group showed a reduction in the total SCFAs and in the concentration of acetic, isobutyric, butyric, and isovaleric acids after scGOS/lcFOS supplementation [13]. These differences could be explained by the fact that, in the present study, we took into consideration the weight of dry cecal content after 12 h in a stove and the amount of water used in the previous homogenization to avoid the effect of the type of supplement in the quantification of the SCFAs. In fact, in a recent paper, using this approach in a non-infection model, the interventions did not affect the cecal levels of SCFA [23].

## 5. Conclusions

In conclusion, the supplementation of suckling rats with scGOS/lcFOS, Lactofidus^TM^, and their combination prevented and ameliorated the diarrhea caused by RV. All supplementations showed changes in the immunoglobulin profile, intestinal gene expression, and gut microbiota. scGOS/lcFOS and Lactofidus^TM^ seem to ameliorate the diarrhea through different mechanisms. The prebiotic mixture reduced gut permeability and changed the SCFA profile. On the other hand, the postbiotic enhanced the expression of TLRs and reduced pH in feces. The combination of scGOS/lcFOS and Lactofidus™ kept most individually observed effects and even showed effects that were not seen separately (i.e., increase in white blood cells and lymphocytes recruitment and upregulation of TLR7 and TLR9 gene expression). The use of both products has been a good strategy to improve the prevention and treatment in the early life rotavirus induced diarrhea rat model. Thus, this combination could be suggested as a good candidate to be used in infants’ formulas for this purpose.

## Figures and Tables

**Table 1:** Growth-associated measurements and weight of organs, normalized by body weight (BW) at the peak of diarrhea (day 8 of life).

	REF	RV	RV + PRE	RV + POST	RV + P/P
**Growth measurements**					
Naso-anal length (Body, cm)	6.82 ± 0.11	6.95 ± 0.09	6.97 ± 0.05	7.08 ± 0.11	6.91 ± 0.09
Anus-tail length (Tail, cm)	3.04 ± 0.03	3.06 ± 0.05	3.03 ± 0.04	3.12 ± 0.06	2.92 ± 0.07
Naso-tail length (cm)	9.83 ± 0.13	10.01 ± 0.11	10.00 ± 0.08	10.20 ± 0.15	9.83 ± 0.13 ^Δ^
Body/Tail length ratio	2.23 ± 0.02	2.28 ± 0.04	2.31 ± 0.03	2.28 ± 0.04	2.38 ± 0.06 *
Body mass index (g/cm^2^)	0.24 ± 0.01	0.25 ± 0.01	0.24 ± 0.01	0.27 ± 0.00 *^ϕ^	0.25 ± 0.01
Lee index (g^0.33^/cm, ×1000)	329.65 ± 3.05	331.79 ± 3.75	325.54 ± 2.29 ^#^	334.63 ± 2.56 ^ϕ^	329.91 ± 4.25
**Weight of organs**					
Spleen/BW ratio (%)	0.56 ± 0.03	0.53 ± 0.05	0.62 ± 0.04	0.73 ± 0.05 *^#^	0.69 ± 0.03 *^#^
Thymus/BW ratio (%)	0.32 ± 0.02	0.29 ± 0.02	0.26 ± 0.01 *	0.32 ± 0.01 ^ϕ^	0.26 ± 0.01 *^Δ^
Liver/BW ratio (%)	2.99 ± 0.05	2.90 ± 0.08	2.75 ± 0.09	3.09 ± 0.07 ^ϕ^	3.06 ± 0.05 ^ϕ^
Large intestine ^1^/BW ratio (%)	0.43 ± 0.01	0.42 ± 0.02	0.49 ± 0.01	0.41 ± 0.02 ^ϕ^	0.43 ± 0.02
Small intestine ^2^/BW ratio (%)	3.26 ± 0.07	3.45 ± 0.13	4.86 ± 0.11 *^#^	3.51 ± 0.10 *^ϕ^	5.09 ± 0.13 *^#Δ^
Large intestine length/BW (cm/g)	44.43 ± 1.62	40.86 ± 1.37	39.13 ± 0.04 *	36.47 ± 1.29 *^#^	39.98 ± 0.93 ^Δ^
Small intestine length/BW (cm/g)	233.68 ± 8.80	227.92 ± 7.36	239.00 ± 0.01	211.55 ± 7.04 ^ϕ^	243.62 ± 6.04 ^Δ^
Stomach/BW ratio (%)	0.70 ± 0.02	0.66 ± 0.02	0.74 ± 0.11	0.67 ± 0.02	0.70 ± 0.02

Relative weight of organs was expressed as percentage (%), with respect to the body weight (BW), and growth-associated measurements are expressed as mean ± SEM (*n* = 12 animals/group). Statistical significance: * *p* < 0.05 RV vs. REF, ^#^ *p* < 0.05 vs. RV, ^ϕ^ vs. RV + PRE, and ^Δ^ vs. RV + POST. REF: reference group; RV: rotavirus group; RV + PRE: rotavirus group, supplemented with a mixture of scGOS and lcFOS; RV + POST: rotavirus group supplemented with Lactofidus^TM^; RV + P/P: rotavirus group supplemented with the combination of both. ^1^ Large intestine weight includes the intestinal content. ^2^ Small intestine was weighted after being rinsed with PBS.

**Table 2:** Clinical variables determining the diarrhea process in the RV groups.

Clinical Outcome	Variable	RV	RV + PRE	RV + POST	RV + P/P
**Incidence**					
	MDA	58.33	39.13	34.78	45.83
	MDAd	8.00	7.00	7.00	7.00
	daAUC	166.67	159.69	86.23	222.92
	MDF	82.35	47.37	50.00	68.75
	MDFd	8.00	7.00	6.00	7.00
	dfAUC	258.35	232.49	146.01	315.60
**Duration**					
	DDB	6.78 ± 0.32	7.22 ± 0.98	7.00 ± 0.33	6.82 ± 0.88
	DDE	8.33 ± 0.24	7.78 ± 1.01	7.63 ± 0.38	8.18 ± 0.87
	DP	1.17 ± 0.34	0.56 ± 0.24	0.42 ± 0.26	1.25 ± 0.49
	DwD	1.75 ± 0.37	1.42 ± 0.31	0.92 ± 0.23	2.25 ± 0.43 ^Δ^
**Severity**					
	MDI	2.39 ± 0.15	2.02 ± 0.09	1.84 ± 0.15 ^#^	2.25 ± 0.10 ^Δ^
	MDId	7.48 ± 0.16	7.27 ± 0.43	7.18 ± 0.23	7.77 ± 0.61
	sAUC	3.73 ± 0.43	2.58 ± 0.23 ^#^	2.58 ± 0.23 ^#^	3.13 ± 0.25

Results are expressed as mean ± SEM (*n* = 12 animals/group). MDA, maximum percentage of diarrheic animals; MDAd, day with maximum percentage of diarrheic animals; daAUC, area under the curve of diarrheic animals no normalized; MDF, maximum percentage of diarrheic feces; MDFd, day with maximum percentage of diarrheic feces; dfAUC, area under the curve of diarrheic feces no normalized. DDB, day of diarrhea beginning (DPI); DDE, day of diarrhea ending (DPI); DP, diarrhea period; DwD, days with diarrhea. MDI, maximum diarrhea index; MDId, day of maximum diarrhea index (DPI); sAUC, area under the curve of severity no normalized. Statistical differences: ^#^ vs. RV, ^Δ^ vs. RV + POST.RV: rotavirus group; RV + PRE: rotavirus group supplemented with a mixture of scGOS/lcFOS; RV + POST: rotavirus group supplemented with Lactofidus^TM^; RV + P/P: rotavirus group supplemented with the combination of both.

**Table 3:** SCFA production in cecal samples at the end of the study (day 16).

	REF	RV	RV + PRE	RV + POST	RV + P/P
SCFA (µmol/g)	29,019.90 ± 4043.90	25,353.06 ± 2889.38	30,680.63 ± 2873.36	27,726.29 ± 3082.71	39,014.80 ± 7066.49
Acetic acid (%)	89.26 ± 1.29	89.35 ± 1.59	97.00 ± 0.52 *^#^	91.47 ± 1.12 ^ϕ^	96.44 ± 0.52 *^#Δ^
Propionic acid (%)	9.36 ± 1.11	9.09 ± 1.37	2.23 ± 0.44 *^#^	6.91 ± 0.96 ^ϕ^	1.71 ± 0.29 *^#Δ^
Isobutyric acid (%)	0.28 ± 0.04	0.19 ± 0.03	0.08 ± 0.02 *^#^	0.22 ± 0.03 ^ϕ^	0.16 ± 0.04
Butyric acid (%)	0.86 ± 0.17	1.12 ± 0.32	0.56 ± 0.17	1.14 ± 0.28 ^ϕ^	1.50 ± 0.36 ^ϕ^
Isovaleric acid (%)	0.20 ± 0.03	0.19 ± 0.03	0.10 ± 0.01 *^#^	0.21 ± 0.03 ^ϕ^	0.12 ± 0.02 ^Δ^
Valeric acid (%)	0.06 ± 0.02	0.07 ± 0.01	0.10 ± 0.02	0.05 ± 0.01	0.11 ± 0.02 *^Δ^

Results are represented as mean ± SEM (*n* = 12 samples/group). Total SCFA are expressed as µmol/g of dry feces Statistical differences: * vs. REF, ^#^ vs. RV, ^ϕ^ vs. RV + PRE, ^Δ^ vs. RV + POST. REF: reference group; RV: rotavirus group; RV + PRE: rotavirus group supplemented with a mixture of scGOS/lcFOS; RV + POST: rotavirus group supplemented with Lactofidus^TM^; RV + P/P: rotavirus group supplemented with the combination of both.

## Data Availability

The datasets generated and/or analyzed during the current study are available from the corresponding author on reasonable request.

## Supplementary Materials

The following supporting information can be downloaded at: https://www.mdpi.com/article/10.3390/nu14061163/s1, Figure S1: Fecal pH) and stomach content pH in diarrhea and post-diarrhea periods; Table S1: Growth-associated measurements and relative weight of organs at the end of the study (day 16 of life); Table S2: Hematological variables at the end of the study (day 8); Table S3: Hematological variables at the end of the study (day 16), Table S4: Clinical variables determining the diarrhea process in the RV groups; Table S5: Relative proportion of the minority families and genres of the gut microbiota with statistical significance among groups without normalization process.
