# Supplementary material for: Preventive Effect of a Postbiotic and Prebiotic Mixture in a Rat Model of Early Life Rotavirus Induced-Diarrhea
Source: Nutrients. 2022 Mar 10;14(6):1163. doi: 10.3390/nu14061163 (PMC8954028; doi:10.3390/nu14061163)
Supplement: Supplementary file 1 [file nutrients-14-01163-s001.zip › nutrients-1625603-supplementary.pdf]

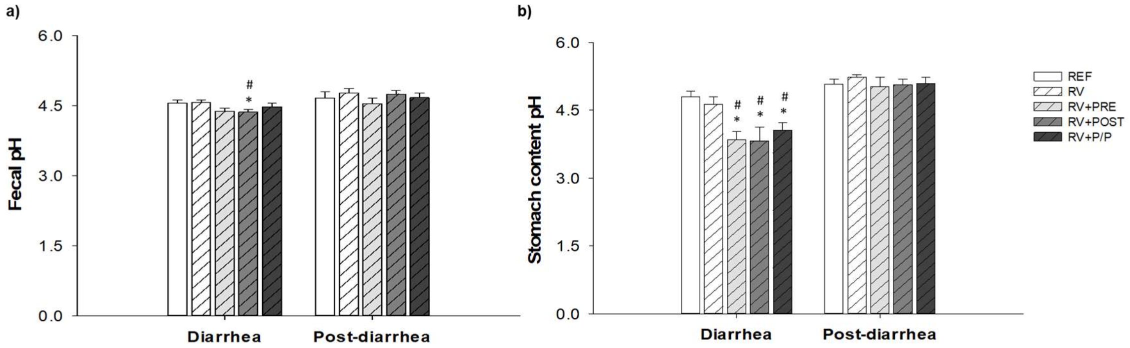

Supplementary Figure S1. Fecal pH (**a**) and stomach content pH (**b**) in diarrhea (on days 7-9 in feces and day 8 in stomach content) and post-diarrhea periods (on days 14-16 in feces and day 16 in stomach content). Results are expressed as mean ± SEM (n = 24 animals/group. Statistical differences: * *vs.* REF, # *vs.* RV, φ *vs.* RV+PRE, Δ *vs.* RV+POST. REF: reference group; RV: rotavirus group; RV+PRE: rotavirus group supplemented with a mixture of scGOS and lcFOS; RV+POST: rotavirus group supplemented with Lactofidus™; RV+P/P: rotavirus group supplemented with the combination of both.

Supplementary Table S1. Growth-associated measurements and relative weight of organs at the end of the study (day 16 of life).

	RV	RV+PRE	RV+POST	RV+P/P
Naso-anal (Body, cm)	9.39 ± 0.08	9.50 ± 0.12	9.72 ± 0.05 $^{*\#}$	9.68 ± 0.08 $^{*\#}$
Anus-tail (Tail, cm)	4.84 ± 0.09	4.91± 0.06	5.04 ± 0.10	4.82 ± 0.10
Naso-tail (cm)	14.23 ± 0.14	14.41 ± 0.15	14.76 ± 0.10 $^{*\#}$	14.5 ± 0.16
Body/Tail length ratio	1.95 ± 0.03	1.94 ± 0.03	1.94 ± 0.04	2.02 ± 0.03
Body mass index (g/cm^2)	0.32 ± 0.01	0.34 ± 0.01	0.34 ± 0.00 $^{\#}$	0.34 ± 0.00 $^{\#}$
Lee index (g$^{0.33}$/cm, x1000)	324.32 ± 3.45	327.91 ± 2.43	328.16 ± 1.32	328.41 ± 2.20
Spleen/BW ratio (%)	0.48 ± 0.03	0.47 ± 0.03	0.52 ±0.02 *	0.54 ± 0.04 *
Thymus/ BW ratio (%)	0.48 ± 0.03	0.46 ± 0.04	0.53 ± 0.03 $^{*\#\varphi}$	0.44 ± 0.01 $^{\Delta}$
Liver/ BW ratio (%)	3.75 ± 0.19	3.62 ± 0.16	3.70 ± 0.17	3.81 ± 0.10 *
Large int./BW ratio (%)	0.62 ± 0.02	0.73 ± 0.04 $^{*\#}$	0.69 ± 0.04 *	0.74 ± 0.01 $^{*\#\Delta}$
Small int./ BW ratio (%)	3.47 ± 0.19	5.22 ± 0.29 $^{*\#}$	3.53 ± 0.17 $^{\varphi}$	5.44 ± 0.12 $^{*\#\Delta}$
Large int. length/ BW (cm/g)	22.22 ± 1.21	21.72 ± 1.55	19.70 ± 0.95 *	18.87 ± 0.64 $^{*\#}$
Small int. length/ BW (cm/g)	134.50 ± 5.47	148.94 ± 8.79	131.54 ± 7.06	133.09 ± 1.92
Stomach/ BW ratio (%)	0.79 ± 0.03	0.81 ± 0.06	0.80 ± 0.05	0.67 ± 0.06

Growth-associated measurements were expressed as mean ± SEM. Relative weight of organs is expressed as percentage (%) with respect to the body weight and (n = 12). Statistical differences: * *vs.* REF, $^{\#}$ *vs.* RV, $^{\varphi}$ *vs.* RV+PRE, $^{\Delta}$ *vs.* RV+POST. REF: reference group; RV: rotavirus group; RV+PRE: rotavirus group supplemented with a mixture of scGOS/lcFOS; RV+POST: rotavirus group supplemented with Lactofidus™; RV+P/P: rotavirus group supplemented with the combination of both.

Supplementary Table S2. Hematological variables at the end of the study (day 8).

	**REF**	**RV**	**RV+PRE**	**RV+POST**	**RV+P/P**
WBC (10^9 cells/L)	2.17 ± 0.36	2.83 ± 0.49	4.09 ± 0.75	4.15 ± 0.76	6.24 ± 0.51 $^{*\# \varphi \Delta}$
LYM (%)	63.35 ± 5.70	65.63 ± 3.49	65.47 ± 2.96	69.15 ± 2.33	72.86 ± 2.73
MID (%)	7.75 ± 0.82	6.45 ± 0.30	7.09 ± 0.52	6.22 ± 0.53	4.99 ± 0.35 $^{\# \varphi}$
GRAN (%)	28.90 ± 2.80	27.93 ± 3.47	27.44 ± 2.80	24.64 ± 2.49	22.15 ± 2.45
LYM (10^9 cells/L)	1.43 ± 0.22	1.79 ± 0.35	2.68 ± 0.55	2.41 ± 0.45	4.46 ± 0.40 $^{*\# \varphi \Delta}$
MID (10^9 cells/L)	0.14 ± 0.02	0.14 ± 0.04	0.23 ± 0.04	0.22 ± 0.04	0.25 ± 0.03 $^{\#}$
GRAN (10^9 cells/L)	0.80 ± 0.14	0.89 ± 0.15	1.18 ± 0.19	1.09 ± 0.21	1.52 ± 0.20 $^{*\#}$
HGB (g/L)	103.67 ± 7.03	76.67 ± 6.23	102.00 ± 13.13 $^{\#}$	82.55 ± 6.42	113.55 ± 7.80 $^{*\#\Delta}$
HCT (%)	25.51 ± 0.49	23.28 ± 1.48	23.45 ± 1.01	25.61 ± 1.76	26.39 ± 0.85 $^{\varphi}$
MCV (fL)	68.68 ± 2.70	96.50 ± 1.16	96.83 ± 0.83	98.55 ± 0.62	100.99 ± 0.59 $^{*\# \varphi \Delta}$
MCH (pg)	28.08 ± 2.53	31.53 ± 1.60	41.13 ± 5.28 $^{\#}$	31.75 ± 1.42 $^{\varphi}$	44.40 ± 4.61 $^{*\#\Delta}$
PLT (10^9 cells/L)	477.58 ± 26.52	227.75 ± 58.78	388.78 ± 55.28 $^{\#}$	288.27 ± 67.51	377.91 ± 44.24
MPV (fL)	9.22 ± 0.32	15.07 ± 1.92 *	10.66 ± 1.41$^{\#}$	11.91 ± 2.00	10.71 ± 0.77

White Blood Cells (WBC), Lymphocyte (LYM), Monocytes, eosinophils, basophils, blasts and other precursor white cells (MID), Neutrophils, monocytes, eosinophils, and basophils (GRAN), Hemoglobin (HGB), Hematocrit (HCT), Mean Cell Volume (MCV), Mean Cell Hemoglobin (MCH), Platelets (PLT) and Mean Platelet Volume (MPV). Results are expressed ad mean ± SEM (n = 12). Statistical differences: * *vs.* REF, $^{\#}$ *vs.* RV, $^{\varphi}$ *vs.* RV+PRE, $^{\Delta}$ *vs.* RV+POST. REF: reference group; RV: rotavirus group; RV+PRE: rotavirus group supplemented with a mixture of scGOS/lcFOS; RV+POST: rotavirus group supplemented with Lactofidus™; RV+P/P: rotavirus group supplemented with the combination of both.

Supplementary Table S3. Hematological variables at the end of the study (day 16).

	RV	RV+PRE	RV+POST	RV+P/P
WBC (10^9 cells/L)	2.59 ± 0.33	2.08 ± 0.38	2.37 ± 0.38	2.37 ± 0.33
LYM (%)	65.43 ± 1.95	66.81 ± 11.66	66.04 ± 1.59	66.07 ± 2.00
MID (%)	7.53 ± 0.53	7.93 ± 1.46	7.68 ± 0.30	6.80 ± 0.32
GRAN (%)	27.03 ± 1.82	25.26 ± 4.50	26.28 ± 1.53	27.13 ± 1.90
LYM (10^9 cells/L)	1.63 ± 0.19	1.34 ± 0.25	1.50 ± 0.27	1.48 ± 0.18
MID (10^9 cells/L)	0.15 ± 0.03	0.11 ± 0.02	0.15 ± 0.04	0.11 ± 0.03
GRAN (10^9 cells/L)	0.68 ± 0.09	0.63 ± 0.12	0.72 ± 0.09	0.78 ± 0.13
HGB (g/L)	98.33 ± 6.93	103.75 ± 19.04	111.42 ± 9.42	90.17 ± 4.50
HCT (%)	25.59 ± 1.63	25.58 ± 4.75	28.53 ± 2.41	23.92 ± 0.64 *
MCV (fL)	65.08 ± 0.92	64.36 ± 11.22	63.82 ± 0.54 *	64.24 ± 0.55 *
MCH (pg)	24.98 ± 1.07	26.19 ± 4.63	25.13 ± 1.40	24.07 ± 0.93
PLT (10^9 cells/L)	454.58 ± 43.04	498.00 ± 91.08	448.00 ± 47.05	479.00 ± 41.26
MPV (fL)	8.64 ± 0.18	8.33 ± 1.46	8.98 ± 0.24	8.95 ± 0.21 $^{\varphi}$

White Blood Cells (WBC), Lymphocyte (LYM), Monocytes, eosinophils, basophils, blasts and other precursor white cells (MID), Neutrophils, monocytes, eosinophils, and basophils (GRAN), Hemoglobin (HGB), Hematocrit (HCT), Mean Cell Volume (MCV), Mean Cell Hemoglobin (MCH), Platelets (PLT) and Mean Platelet Volume (MPV). Results are expressed ad mean ± SEM (n = 12). Statistical differences: * *vs.* REF, $^{\#}$ *vs.* RV, $^{\varphi}$ *vs.* RV+PRE, $^{\Delta}$ *vs.* RV+POST. REF: reference group; RV: rotavirus group; RV+PRE: rotavirus group supplemented with a mixture of scGOS/lcFOS; RV+POST: rotavirus group supplemented with Lactofidus™; RV+P/P: rotavirus group supplemented with the combination of both.

Supplementary Table S4. Clinical variables determining the diarrhea process in the RV groups without normalization process.

Clinical Outcome	Variable	RV	RV+PRE	RV+POST	RV+P/P
**Incidence**					
	MDA	58.33	12.50	34.78	4.17
	MDAd	8.00	9.00	7.00	7.00
	daAUC	166.67	29.53	86.23	8.33
	MDF	82.35	20.00	50.00	7.14
	MDFd	8.00	9.00	6.00	7.00
	dfAUC	258.35	44.91	146.01	12.70
**Duration**					
	DDB	6.78 ± 0.32	7.33 ± 0.88	7.00 ± 0.33	7.00 ± 0.00
	DDE	8.33 ± 0.24	7.33 ± 0.88	7.63 ± 0.38	7.33 ± 0.33
	DP	1.17 ± 0.34	0.00 ± 0.00 $^{\#}$	0.42 ± 0.26	0.09 ± 0.09 $^{\#}$
	DwD	1.75 ± 0.37	0.27 ± 0.14 $^{\#}$	0.92 ± 0.23 $^{\varphi}$	0.36 ± 0.20 $^{\#}$
**Severity**					
	MDI	2.39 ± 0.15	1.52 ± 0.08 $^{\#}$	1.84 ± 0.15 $^{\#\varphi}$	1.43 ± 0.07 $^{\#\Delta}$
	MDId	7.48 ± 0.16	7.79 ± 0.67	7.18 ± 0.23	7.81 ± 0.71
	sAUC	3.73 ± 0.43	0.68 ± 0.13 $^{\#}$	2.58 ± 0.23 $^{\#\varphi}$	0.53 ± 0.14 $^{\#\Delta}$

Results are expressed as mean ± SEM (n = 12 animals/group). MDA, maximum percentage of diarrheic animals; MDAd, day with maximum percentage of diarrheic animals; daAUC, area under the curve of diarrheic animals normalized; MDF, maximum percentage of diarrheic feces; MDFd, day with maximum percentage of diarrheic feces; dfAUC, area under the curve of diarrheic feces normalized. DDB, day of diarrhea beginning (DPI); DDE, day of diarrhea ending (DPI); DP, diarrhea period; DwD, days with diarrhea. MDI, maximum diarrhea index; MDId, day of maximum diarrhea index (DPI); sAUC, area under the curve of severity normalized. Statistical differences: * *vs.* REF, $^{\#}$ *vs.* RV, $^{\varphi}$ *vs.* RV+PRE, $^{\Delta}$ *vs.* RV+POST. REF: reference group; RV: rotavirus group; RV+PRE: rotavirus group supplemented with a mixture of scGOS and lcFOS; RV+POST: rotavirus group supplemented with Lactofidus™; RV+P/P: rotavirus group supplemented with the combination of both.

Supplementary Table S5. Relative proportion of the minority families and genres of the gut microbiota with statistical significance among groups.

	REF	RV	RV+PRE	RV+POST	RV+P/P
**Minority families**					
Caulobacteraceae	0.19 ± 0.05	0.26 ± 0.08	0.20 ± 0.06	$0.03 \pm 0.01^{*\# \varphi}$	$0.04 \pm 0.02^{*\# \varphi}$
Sphingomonadaceae	1.29 ± 0.41	1.33 ± 0.39	2.17 ± 0.95	$0.36 \pm 0.10^{*\#}$	$0.27 \pm 0.06^{*\# \varphi}$
Rhizobiaceae	1.15 ± 0.41	1.40 ± 0.41	1.83 ± 0.90	$0.21 \pm 0.07^{\# \varphi}$	$0.16 \pm 0.04^{*\#\varphi}$
Burkholderiaceae	0.41 ± 0.17	0.38 ± 0.18	0.50 ± 0.25	0.06 ± 0.02	$0.01 \pm 0.01^{*}$
Beijerinckiaceae	0.17 ± 0.06	0.09 ± 0.03	0.08 ± 0.04	-	$0.02 \pm 0.01^{*}$
Erysipelotrichaceae	1.36 ± 0.59	0.43 ± 0.18	0.47 ± 0.27	0.11 ± 0.03	$0.69 \pm 0.15^{\Delta}$
Propionibacteriaceae	0.04 ± 0.02	-	0.02 ± 0.01	-	-
**Minority genres**					
Sphingomonas	1.45 ± 0.43	1.76 ± 0.34	2.26 ± 0.97	$0.36 \pm 0.10^{*\# \varphi}$	$0.29 \pm 0.06^{*\# \varphi}$
Ralstonia	0.39 ± 0.15	0.27 ± 0.12	0.51 ± 0.25	$0.05 \pm 0.02^{*}$	$0.01 \pm 0.01^{*}$
Methylobacterium	0.19 ± 0.03	0.12 ± 0.03	0.08 ± 0.05	-	$0.02 \pm 0.01^{*\#}$
Vibrionimonas	2.72 ± 0.75	3.42 ± 0.47	2.25 ± 0.50	$0.87 \pm 0.24^{\#}$	$1.22 \pm 0.23^{\#}$
Staphylococcus	2.87 ± 0.61	2.19 ± 0.49	$5.44 \pm 1.11^{\#}$	$2.28 \pm 0.83^{\varphi}$	$0.91 \pm 0.23^{* \varphi}$
Mesorhizobium	0.60 ± 0.18	1.31 ± 030	1.53 ± 0.80	$0.19 \pm 0.05^{\# \varphi}$	$0.17 \pm 0.04^{\# \varphi}$
Turicibacter	1.59 ± 0.66	0.64 ± 0.22	0.48 ± 0.27	0.12 ± 0.04	$0.72 \pm 0.14^{\Delta}$
Clostridium stricto sensu 1	1.20 ± 0.47	1.47 ± 0.46	$0.21 \pm 0.08^{\#}$	$0.14 \pm 0.04^{\#}$	$2.12 \pm 1.05^{\varphi \Delta}$
Bifidobacterium	-	-	-	0.46 ± 0.17	0.89 ± 0.20
Cutibacterium	0.04 ± 0.02	-	0.03 ± 0.02	-	-
Methylobacterium	0.188 ± 0.06	0.12 ± 0.03	0.08 ± 0.05	-	0.02 ± 0.01
Phyllobacterium	0.51 ± 0.18	0.52 ± 0.08	0.38 ± 0.14	-	-

Results are expressed as mean ± SEM (n = 6 animals/group). Statistical differences: * *vs.* REF, $^{\#}$ *vs.* RV, $^{\varphi}$ *vs.* RV+PRE, $^{\Delta}$ *vs.* RV+POST. REF: reference group; RV: rotavirus group; RV+PRE: rotavirus group supplemented with a mixture of scGOS and lcFOS; RV+POST: rotavirus group supplemented with Lactofidus™; RV+P/P: rotavirus group supplemented with the combination of both.
